# Opposing effects of β-2 and β-1 adrenergic receptor signaling on neuroinflammation and dopaminergic neuron survival in α-synuclein-mediated neurotoxicity

**DOI:** 10.1186/s12974-023-02748-3

**Published:** 2023-03-02

**Authors:** Daniel Torrente, Enming J. Su, Gerald P. Schielke, Mark Warnock, Kris Mann, Daniel A. Lawrence

**Affiliations:** 1 Department of Molecular and Integrative Physiology, University of Michigan Medical School, 7301 MSRB III, 1150 W. Medical Center Dr., Ann Arbor, MI 48109-0644 USA; 2 Department of Internal Medicine, Division of Cardiovascular Medicine, University of Michigan Medical School, Ann Arbor, MI USA

**Keywords:** α-Synuclein, Substantia nigra, Parkinson disease, Dopaminergic neurons, Propranolol, Clenbuterol, DSP-4, Xamoterol, Microglia, T-cell

## Abstract

**Background:**

Noradrenergic neurons in the locus coeruleus (LC) are the primary source of norepinephrine (NE) in the brain and degeneration of these neurons is reported in the early stages of Parkinson’s disease (PD), even prior to dopaminergic neuron degeneration in the substantia nigra (SN), which is a hallmark of PD pathology. NE depletion is generally associated with increased PD pathology in neurotoxin-based PD models. The effect of NE depletion in other models of PD-like α-synuclein-based models is largely unexplored. In PD models and in human patients, β-adrenergic receptors’ (AR) signaling is associated with a reduction of neuroinflammation and PD pathology. However, the effect of NE depletion in the brain and the extent of NE and β-ARs signaling involvement in neuroinflammation, and dopaminergic neuron survival is poorly understood.

**Methods:**

Two mouse models of PD, a 6OHDA neurotoxin-based model and a human α-synuclein (hα-SYN) virus-based model of PD, were used. DSP-4 was used to deplete NE levels in the brain and its effect was confirmed by HPLC with electrochemical detection. A pharmacological approach was used to mechanistically understand the impact of DSP-4 in the hα-SYN model of PD using a norepinephrine transporter (NET) and a β-AR blocker. Epifluorescence and confocal imaging were used to study changes in microglia activation and T-cell infiltration after β1-AR and β2-AR agonist treatment in the hα-SYN virus-based model of PD.

**Results:**

Consistent with previous studies, we found that DSP-4 pretreatment increased dopaminergic neuron loss after 6OHDA injection. In contrast, DSP-4 pretreatment protected dopaminergic neurons after hα-SYN overexpression. DSP-4-mediated protection of dopaminergic neurons after hα-SYN overexpression was dependent on β-AR signaling since using a β-AR blocker prevented DSP-4-mediated dopaminergic neuron protection in this model of PD. Finally, we found that the β-2AR agonist, clenbuterol, reduced microglia activation, T-cell infiltration, and dopaminergic neuron degeneration, whereas xamoterol a β-1AR agonist showed increased neuroinflammation, blood brain barrier permeability (BBB), and dopaminergic neuron degeneration in the context of hα-SYN-mediated neurotoxicity.

**Conclusions:**

Our data demonstrate that the effects of DSP-4 on dopaminergic neuron degeneration are model specific, and suggest that in the context of α-SYN-driven neuropathology, β2-AR specific agonists may have therapeutic benefit in PD.

**Supplementary Information:**

The online version contains supplementary material available at 10.1186/s12974-023-02748-3.

## Background

Parkinson’s disease (PD) is characterized by the abnormal accumulation of α-synuclein (α-Syn) in neuronal cell bodies and the progressive loss of dopaminergic neurons in the substantia nigra *pars compacta* (SNpc). Besides dopaminergic neurons, degeneration of other neuronal populations is known to contribute to PD symptoms and pathology, including noradrenergic neurons located in the locus coeruleus (LC) [1]. LC-Noradrenergic neurons are the main source of norepinephrine (NE) in the brain and degeneration of these neurons is reported to precede dopaminergic neuron loss in PD patients [1–3]. However, the role of the NE system in PD pathology and in dopaminergic neuron survival is poorly understood.

The outcomes resulting from perturbations of the NE system in the central nervous system (CNS) and in PD pathology are complex. For instance, in neurotoxin-based models of PD, reduction of brain NE is shown to increase neuroinflammation and dopaminergic neuron degeneration in the SNpc [4–6], while other studies find no effect on dopamine levels or dopaminergic neuron survival after brain NE depletion [7–10]. Outside of PD pathologies, depletion of brain NE has been reported to be neuroprotective by reducing microglia activation [11]. These diverse responses reflect the many and complex physiological functions regulated by NE in the brain [12, 13]. NE signaling is primarily driven by different G protein-coupled receptors, such as α-1, α-2, β-1, and β-2 adrenergic receptors (AR) which are proposed to regulate different functions in the CNS, such as wakefulness, attention, and neuroinflammation [12–14]. In PD, a special clinical interest is focused on β-ARs signaling, since PD animal models and human patients show a link between promoting β-ARs signaling and a reduction of PD risk and pathology, whereas blocking β-ARs is associated with increased PD risk [15–18]. However, the mechanisms associated with NE and β-ARs signaling on dopaminergic neuron survival is still elusive.

The selective noradrenergic neurotoxin, DSP-4, is the most common and widely accepted approach to deplete NE levels in the CNS [19]. The correlation between brain NE depletion by DSP-4 and increased dopaminergic neuron degeneration in the SNpc is mainly founded on neurotoxin-based models of PD [4–6], while other animal models representing different pathological features of PD have not been extensively studied. In the present work, we explored the effects of NE depletion in the CNS using DSP-4 in a human α-SYN (hα-SYN) viral-based mouse model of PD with the goal to identify novel therapeutic targets that involve the NE system in the context of hα-SYN-mediated neurotoxicity.

## Materials and methods

### Mice

Male WT C57BL/6J mice between 8 and 14 weeks were used in all experiments and were obtained from Jackson Laboratories. Mice were housed under a 12-h light/dark cycle with free access to water and standard rodent chow. All animal procedures were approved by and carried out in accordance with the guidelines of the Institutional Animal Care and Use Committee at the University of Michigan.

### Stereotaxic 6OHDA injection

Mice were anesthetized with 2% isoflurane and secured on a stereotactic frame. 6-Hydroxydopamine (6OHDA; 2547; Tocris) was dissolved in a 0.2% ascorbic acid saline solution. A 0.2% ascorbic acid saline solution was used as vehicle control. WT mice received a unilateral injection of 6OHDA or vehicle control in the striatum. The striatum coordinate used for stereotaxic injection was AP = 0.7 ML = − 2.0 DV = − 2.0 mm. A total of 5 μg of 6OHDA in 2 μL was injected in the striatum at a rate of 0.5 μL/min with a 33G nanofil blunt needle (NF33BL-2, World Precision Instruments) connected to FEP tubbing and a 25 μL Hamilton syringe. After 6OHDA infusion, the nanofil blunt needle was left in place for 5 min. Four weeks after injection, mice were PBS and 4% PFA perfused for immunohistochemistry analysis.

### rAAV2–hα-SYN design and production

Recombinant adeno-associated virus serotype 2 (rAAV2) expressing human wild-type α-SYN (rAAV2–hα-SYN) or rAAV2-empty (rAAV2-control) virus were produced at the University of Michigan Vector core. Cloning of hα-SYN was done using the pAAV–hα-SYN WT plasmid as a template (#36055, Addgene) and the pAAV-CBA plasmid as the vector backbone (#81008; Addgene). The resulting plasmid pAAV–CBA–hα-SYN was used to produce rAAV2–hα-SYN virus, where hα-SYN expression is driven by the chicken-beta actin (CBA) promoter. The final titers of the viral stocks were determined by qPCR and ranged between 2.0 × 10^13^ and 3.12 × 10^13^ viral genome copies per milliliter (vg/mL; plasmids and viruses are available at the University of Michigan Vector core).

### Stereotaxic rAAV2–hα-SYN injection in SN

Mice were anesthetized with 2% isoflurane and secured on a stereotactic frame. WT mice received a unilateral injection of rAAV2–hα-SYN at a final concentration of 2 × 10^13^ vg/mL into the SN. The SN coordinate used for stereotaxic injection was AP = − 3.1 ML = − 1.4 DV = − 4.2 mm. A total of 2 μL of rAAV2–hα-SYN or rAAV2-control virus was injected in the SN at a rate of 0.25 μL/min with a 33G nanofil blunt needle (NF33BL-2, World Precision Instruments) connected to FEP tubbing and a 25 μL Hamilton syringe. After rAAV2–hα-SYN or rAAV2-control virus infusion, the nanofil blunt needle was left in place for 5 min. 4 weeks after injection, mice were PBS and 4% PFA perfused for immunohistochemistry analysis.

### Drug treatments

*N*-(2-chloroethyl)-*N*-ethyl-2-bromobenzylamine hydrochloride (DSP-4) was prepared in saline solution and injected IP in a single dose of 50 mg/kg (2958; Tocris). This dose was selected based on previous studies showing tissue NE depletion in the brain [19]. Three days after DSP-4 treatment, stereotaxic injection of 6OHDA or rAAV2–hα-SYN injection was performed as described above. To block the norepinephrine transporter (NET), we use Desipramine (D3900; Sigma). A single dose of Desipramine (25 mg/kg in saline solution) was delivered IP 45 min prior to DSP-4 treatment. Propranolol was delivered IP daily at 10 mg/kg in saline solution (0624; Tocris). Propranolol treatment started 45 min after stereotaxic injection of rAAV2–hα-SYN injection and was given once a day until the end of the experiment. Xamoterol (3 mg/kg; IP) (24,267; Cayman) and Clenbuterol (10 mg/kg; IP) (C5423, Sigma) daily treatment was performed beginning 45 min after stereotaxic injection of rAAV2–hα-SYN injection for 4 weeks until the end of the experiment. All drugs used here were dissolved fresh using saline solution the day of treatment and saline solution was used as vehicle control in all experimental conditions. Clenbuterol and xamoterol doses were selected based on previous studies related to CNS pathologies [16, 20]**.**

### NE and serotonin measurements

The SN and cortex of WT mice 4 weeks after saline, DSP-4 or Desipramine + DSP-4 treatments were dissected and snap-frozen in liquid nitrogen. Then, samples were sonicated on ice by probe sonication on setting 3 with a 30% duty cycle in ice-cold 0.1 M PCA containing 0.1 mM EDTA. Samples were centrifuged at 10,000×*g* for 10 min at 4 °C. Then, supernatants were transferred into fresh 0.22 µM PVDF microcentrifuge filter tubes. NE and serotonin concentrations were determined by reverse-phase high-performance liquid chromatography (HPLC) with electrochemical detection (HPLC-ED). For HPLC, an ESA 5600A CoulArray detection system, equipped with an ESA Model 584 pump and an ESA 542 refrigerated autosampler was used. Separations were performed at 26 °C using an MD-150 × 3.2 mm C18 column. The mobile phase consisted of 1.6 mM 1-octane sulfonic acid sodium, 75 mM NaH2PO4, 0.025% triethylamine, and 8% acetonitrile at pH 3.0. The samples were eluted isocratically at 0.4 mL/min and detected using a 6210 electrochemical cell (ESA, Bedford, MA) equipped with a 5020-guard cell. Guard cell potential was set at 600 mV, while analytical cell potentials were − 175, 150, 350 and 425 mV. The total run time for each run is 40 min. The analytes were identified by the matching criteria of retention time to known standards (Sigma Chemical Co., St. Louis MO). NE and serotonin were quantified by comparing peak areas to those of standards on the dominant sensor. Data were normalized by protein concentration in each brain sample.

### Immunohistochemistry

For immunofluorescence, mice were PBS and 4% PFA-perfused, brains were harvested, post-fixed in PFA, dehydrated in 30% sucrose and embedded in OCT. 14 μm-thick frozen sections were permeabilized and blocked in 0.5% TritonX-100 and 5% Bovine Serum Albumin in 0.5% TritonX-100/1X PBS, respectively. Sections were incubated with primary antibodies in blocking solution overnight at 4 °C followed by incubation with 488, 564 and 647 Alexa-Fluor conjugated secondary antibodies for 1 h at room temperature. After secondary antibody incubations, sections were washed with 0.1% NP-40/1X PBS. DAPI was used to detect cells nuclei. Sections were mounted using ProLong™ Diamond Antifade Mountant (P36961; Life Technologies). The primary antibodies used were: Tyrosine Hydroxylase (TH) (1:1000; ab113, Abcam), hα-SYN (1:300; ab138501, Abcam), TMEM119 (1:500; ab209064, Abcam), CD16/32 (1:100; #101301, BioLegend) and CD3 (1:200; 100347, BioLegend). For staining involving hα-SYN, heat-mediated antigen retrieval was performed using DAKO retrieval solution (S1700; DAKO). All images were acquired with a Nikon Ti-E Eclipse Microscope using a 20× objective (Plan-Apo, 0.75 numerical aperture) or a 60× oil objective (Plan-Apo, 1.4 numerical aperture). Confocal images were acquired using CREST X-Light V2 Spinning Disk. Z-stacks of 60× images were collected at 0.5 μm increments with a total thickness of ~ 6 μm. Images were captured with an ORCA-fusion camera (C14440-20UP, Hamamatsu) or an Andor Zyla camera (Zyla 4.2 sCMOS, Oxford Instruments). Large scan function with Z step focus was used to capture stitched images of the SN using a 20× objective. Confocal images are shown as maximum intensity projection images. Images are representative of the respective staining and were processed and analyzed using NIS-elements advanced research software (Nikon Instrument Inc) and FIJI-Image J open software.

### Quantification of dopaminergic neurons

Estimation of dopaminergic neuron degeneration in the SN was performed by counting the total number of TH^+^ neurons in the injected and uninjected SN 4 weeks after rAAV2–hα-SYN or rAAV2-control. 14 µm-thick coronal brain sections were sampled at intervals of 112 µm through the rostrocaudal extent of the SN, at least four sections per mouse were counted. The total number of TH^+^ neurons (Nt) in the injected and uninjected hemisphere of the SN were estimated using model-based stereology incorporating the Abercrombie correction with the following formula: Nt = Ns × (St/Ss) × *M*/(*M* + *D*), where Ns is the total number of neuron counted, St is the total number of sections in the brain region, Ss is the number of sections sampled, *M* is the thickness of the section, and *D* is the average diameter of the counted neurons [21]. The average diameter of TH neurons was calculated averaging the min and max Feret diameter. Nt for the uninjected SN was used as an internal control in every mouse and a % of dopaminergic neuron survival was obtained using the following formula: TH^+^ neuron survival (%) = (Nt_injected SN_/Nt_uninjected SN_) × 100. The genotype of mice was unknown to the investigator at the time of quantification.

### Fluorescence intensity quantification

Fluorescence intensity of TMEM119, CD16/32 and mouse IgG in the injected and uninjected SN 4 weeks after rAAV2–hα-SYN virus injection was performed as follows: 14 µm-thick coronal brain sections co-stained with TH, TMEM119 and CD16/32 were sampled on intervals of 112 µm through the rostrocaudal extent of the SN, 4 sections per mouse were quantified. A region of interest defining the entire SN *pars compacta* (SNpc) using TH co-staining was used to quantify the sum fluorescence intensity above background in the injected and uninjected SN. The sum fluorescence intensity was normalized to the area of the region of interest. The fold change was calculated by dividing the sum fluorescence intensity of the injected SN by the sum fluorescence intensity of the uninjected SN from each individual mouse. For the average number of CD3^+^ cells quantification in the SN 4 weeks after rAAV2–hα-SYN injection, 14 µm-thick coronal brain sections were stained with CD3 and TH. The average number of CD3+ cells associated with the SNpc were counted by defining the SNpc using TH staining and the average number of CD3+ cells were then estimated in the injected and uninjected (rarely observed) SN in four sections per mouse sampling the rostrocaudal extent of the SN.

### Corridor task

Lateralized sensory-motor integration was measured using a corridor task. This behavioral test was selected due to its sensitivity to detect partial unilateral dopaminergic neuron damage in rats and mouse PD models [22, 23], which is ideal for the mild unilateral degeneration of dopaminergic neurons observed in the SN after rAAV2–hα-SYN injection. This test consists in a long narrow rectangular plexiglass corridor with the following dimensions: *L* = 60 cm × *W* = 4 cm × *H* = 15 cm (testing corridor). The testing corridor contains 10 pairs of adjacent Eppendorf caps placed at 5-cm intervals with 4–5 sugar pellets each (20 mg; TestDiet). A corridor without Eppendorf caps with the same dimensions as the testing corridor was used as the habituation corridor. WT mice treated with clenbuterol or saline daily after rAAV2–hα-SYN virus injection were habituated 1 day before the end of the experiment (4 weeks after injection) in the habituation corridor by scattering sugar pellets along the corridor floor and allowing them to freely explore for 10 min. Lateralized sensory-motor integration was tested 4 weeks after rAAV2–hα-SYN injection. On the testing day, mice were placed in the habituation corridor for 5 min in the absence of sugar pellets; then, mice were transferred to one end of the testing corridor containing sugar pellets and video recorded for 5 min. The video recordings were analyzed by an investigator blinded to the treatment and genotype. The number of ipsilateral and contralateral explorations relative to the injected hemisphere was counted until the mouse made a total of 20 explorations or the video ended. An exploration was defined as a nose-poke into an Eppendorf cap, whether the sugar pellet was poked or eaten, and a new exploration was only counted by exploring a new cap. Data are expressed as a percentage of bias lateralized explorations, calculated as: Bias (%) = 100*(ipsilateral − contralateral)/(ipsilateral + contralateral).

### Statistics

Data analysis was performed using GraphPad Prism 8 statistical software (GraphPad Software, La Jolla, CA, USA). All experiments were repeated at least two independent times and n indicates the number of individual mice used in the study. For statistical analysis, in any experiment with only two groups, a two-tailed t test was used. For experiments with more than two groups, a one-way ANOVA with Tukey or Dunnet post hoc test was used. Data are represented as mean values ± S.E.M; *p* < 0.05 was considered significant.

## Results

### DSP-4 pretreatment increases dopaminergic neuron degeneration in the 6OHDA model of PD

**Fig. 1:**
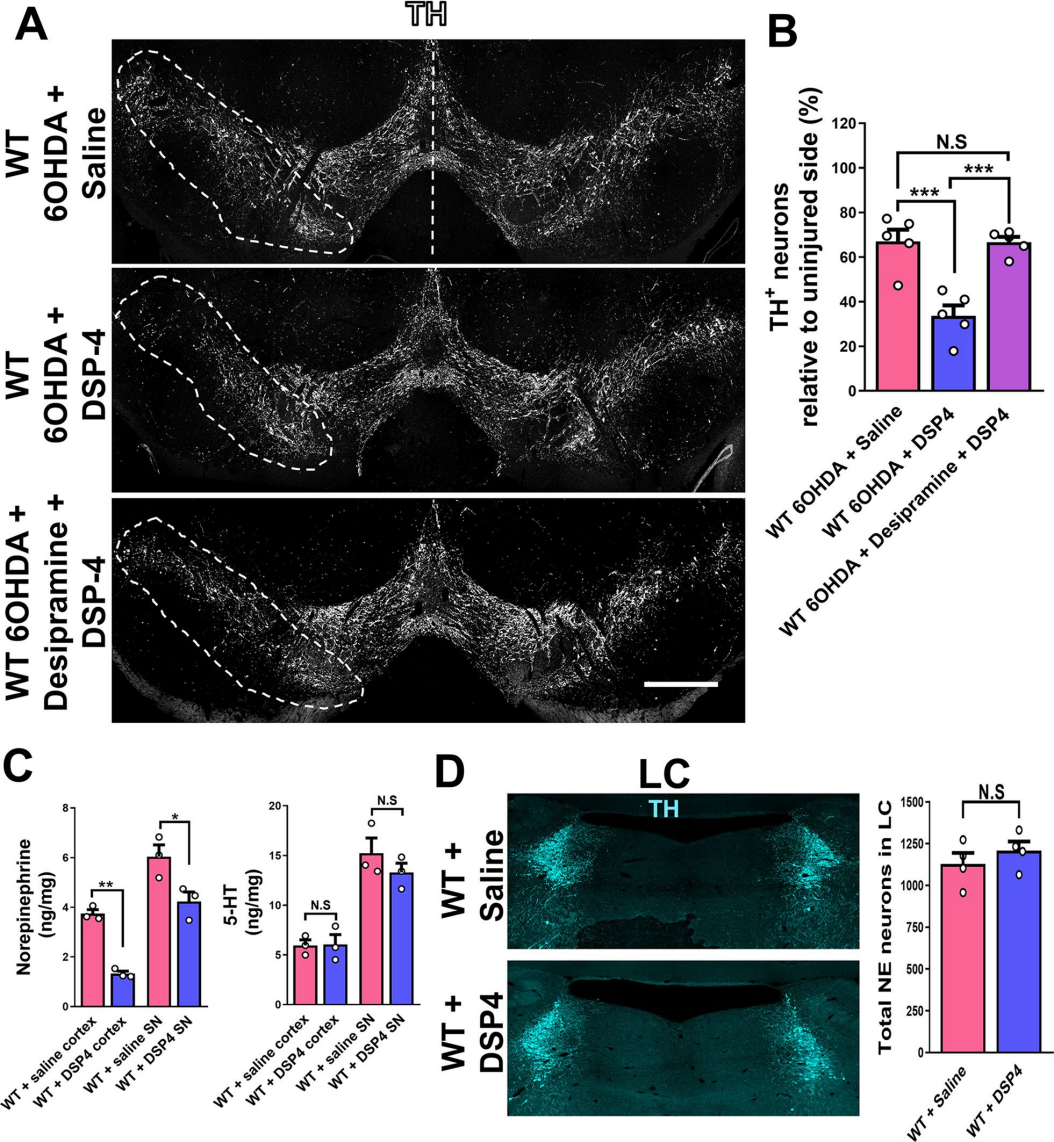
DSP-4 treatment increases dopaminergic neuron degeneration in the 6OHDA model of PD. **A** Representative images of the SN showing dopaminergic neurons degeneration in the injected SN (TH, white) and **B** quantification of TH+ neurons in coronal sections of the SN 4 weeks after 6OHDA injection of WT mice treated with Saline, DSP-4 or Desipramine + DSP-4. **C** NE and serotonin tissue levels measured by HPLC-ED in the cortex and SN of WT mice 4 weeks after DSP-4 or saline treatment. **D** Representative images of the LC showing noradrenergic neurons (TH, cyan) and quantification of TH+ noradrenergic neurons in coronal sections of the LC 4 weeks after DSP-4 or saline injection in WT mice. Data are shown as mean values ± SEM, each dot represents one mouse, N.S: not significant, *p < 0.05; **p < 0.01; ***p < 0.001. One-way ANOVA followed by Tukey post hoc test; two-tailed *t* test. Scale bar = 500 µm

Based on the conflicting results obtained by others using DSP-4 in neurotoxin-based models of PD, DSP-4 treatment either enhanced dopaminergic neuron degeneration [4–6] or failed to disrupt dopamine levels in the brain [24]. We first wanted to confirm the effects of reducing brain NE levels on dopaminergic neuron survival by DSP-4 pretreatment (50 mg/kg; IP) in the 6OHDA neurotoxin-based mouse model of PD. Consistent with previous reports [4–6], DSP-4 pretreatment in WT mice showed a significant increase in dopaminergic neuron degeneration compared to saline-treated WT mice 4 weeks after striatal 6OHDA injection (Fig. 1A, B). DSP-4 is proposed to lead to NE depletion in the brain by first interacting with the norepinephrine transporter (NET) in noradrenergic terminals [19]. To test whether DSP-4-mediated degeneration of dopaminergic neurons in the 6OHDA model of PD is dependent on DSP-4 interaction with NET, we use desipramine, a reversible NET blocker [25]. Desipramine treatment (25 mg/kg; IP) 45 min prior to DSP-4 treatment in WT mice showed a significant reduction in dopaminergic neurons degeneration compared to only DSP-4-treated WT mice 4 weeks after 60HDA injection (Fig. 1A, B). As a control for the injection of 6OHDA, WT mice were injected with vehicle into the striatum which did not show any effect on dopaminergic neuron degeneration (Additional file 1: Fig. S1).

To confirm the effect of DPS-4 in brain NE levels, we measure NE tissue levels in the SN and cortex of WT mice using high-performance liquid chromatography with electrochemical detection (HLPC-ED) 4 weeks after DSP-4 treatment. In WT mice, DSP-4 treatment led to a reduction in NE levels in tissue extracts obtained from the SN and cortex. No effect was observed in serotonin levels after DSP-4 treatment in the SN or cortex (Fig. 1C). Recent reports indicate that NE tissue level reduction after DSP-4 treatment is not associated with noradrenergic neuron degeneration in the LC at early timepoints (less than 3 months after DSP-4 injection) [26]. To test whether reduction of NE tissue levels in the SN and cortex correlates with noradrenergic neuron loss in the LC 4 weeks after DSP-4 treatment, we stained coronal sections of the LC and quantified TH+ neurons in the LC. Consistent with previous reports, no significant reduction in TH+ noradrenergic neurons cells bodies was detected 4 weeks after injection of DSP-4 (Fig. 1D). Overall, these data confirm previous reports of DSP-4 promoting dopaminergic neuron degeneration and suggest that DSP-4 interaction with NET is necessary to induce dopaminergic neuron degeneration in the context of 6OHDA-mediated damage.

### DSP-4 treatment protects dopaminergic neurons degeneration in the SN from hα-SYN-mediated degeneration

**Fig. 2:**
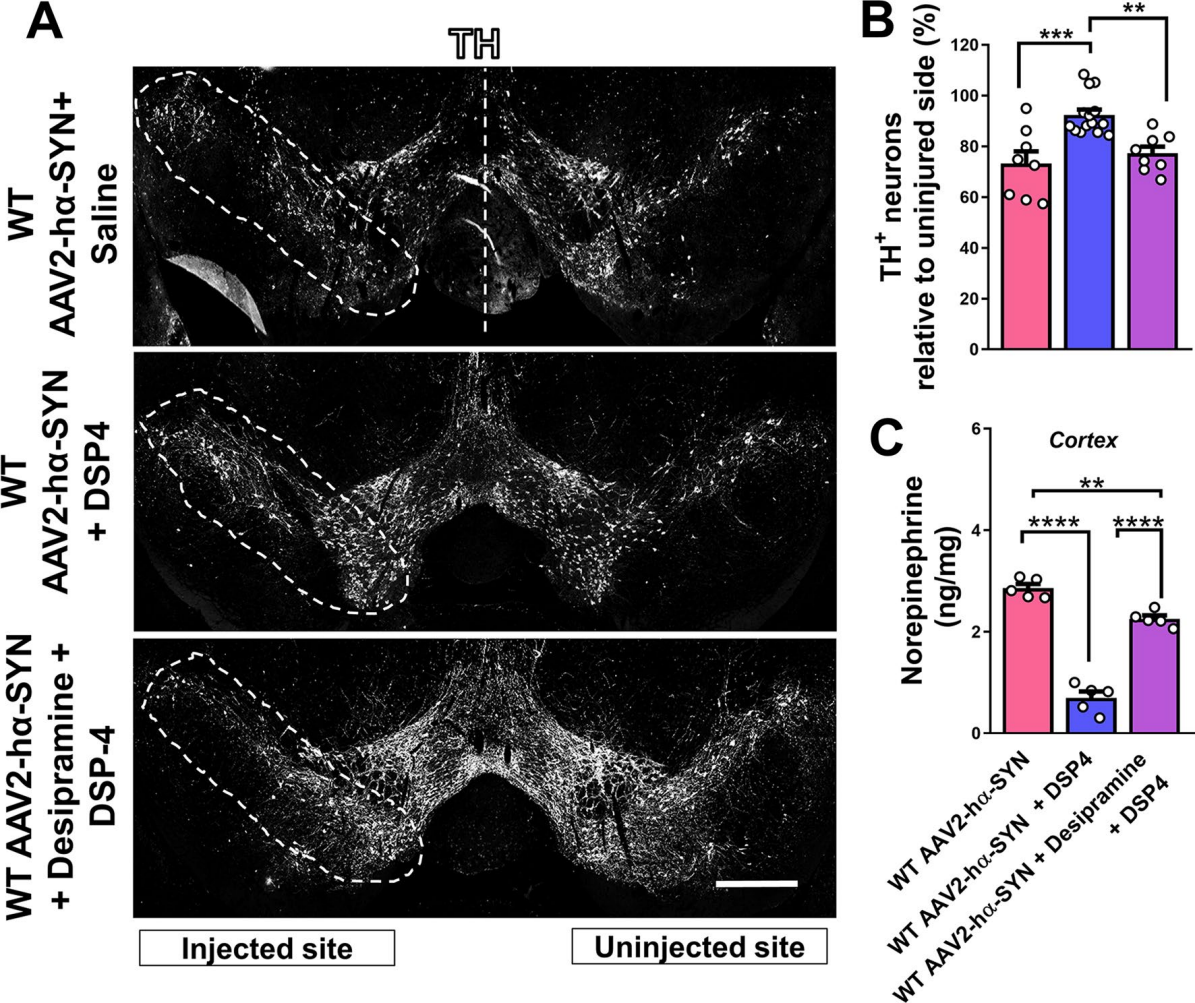
DSP-4 treatment protects dopaminergic neurons degeneration in the SN from hα-SYN-mediated degeneration. **A** Representative images of the SN showing dopaminergic neurons degeneration in the injected SN (TH, white) and **B** quantification of TH+ dopaminergic neurons in coronal sections of the SN 4 weeks after rAAV2–hα-SYN injection in WT mice pretreated with Saline, DSP-4 or Desipramine+ DSP-4. **C** NE tissue levels measured by HPLC-ED in the cortex of WT mice 4 weeks after DSP-4, Desipramine + DSP-4 or saline treatment. Data are shown as mean values ± SEM, each dot represents one mouse, N.S: not significant, **p < 0.01; ***p < 0.001; ****p < 0.0001. One-way ANOVA followed by Tukey post hoc test. Scale bar = (A) 500 µm

We showed that DSP-4 pretreatment promotes dopaminergic neuron degeneration in the 6OHDA neurotoxin-based mouse model of PD. Next, we tested whether reduction of NE tissue levels in the brain using DSP-4 will have the same effects on dopaminergic neuron survival in a hα-SYN mouse model of PD, where overexpression of hα-SYN is achieved using a rAAV2 virus (rAAV2–hα-SYN) (Additional file 1: Fig. S2A). As a control for virus injection, mice were injected with an empty rAAV2-virus into the SN which did not show any significant effect on dopaminergic neuron survival (Additional file 1: Fig. S1). Surprisingly, DSP-4 pretreatment (50 mg/kg; IP) in WT mice showed significant protection of dopaminergic neurons compared to saline-treated WT mice 4 weeks after hα-SYN overexpression (Fig. 2A–C). This result was opposite to the observed effect of DSP-4 in the 6OHDA mouse model of PD (Fig. 1A, B). In addition, desipramine pretreatment (25 mg/kg; IP) in DSP-4-treated WT mice prevented the protection of dopaminergic neurons in the SN compared to only DSP-4-treated WT mice 4 weeks after hα-SYN overexpression (Fig. 2A, B). We confirmed that desipramine pretreatment blocked the reduction of NE tissue levels after DSP-4 treatment in WT mice 4 weeks after DSP-4 injection using HPLC-ED (Fig. 2C). To discard the possibility that DSP-4-mediated protection was due to lack of hα-SYN expression in dopaminergic neurons, we confirmed the presence of hα-SYN in dopaminergic neurons following desipramine pretreatment using an antibody specific for hα-SYN (Additional File 1: Fig. S2). These data suggest that DSP-4-mediated protection of dopaminergic neurons is NET-dependent and not the result of an off-target effect of DSP-4 facilitating dopaminergic neuron protection in the context of hα-SYN-mediated neurotoxicity.

### Blocking β-adrenergic receptors inhibits DSP-4-mediated dopaminergic neurons protection after hα-SYN overexpression

**Fig. 3:**
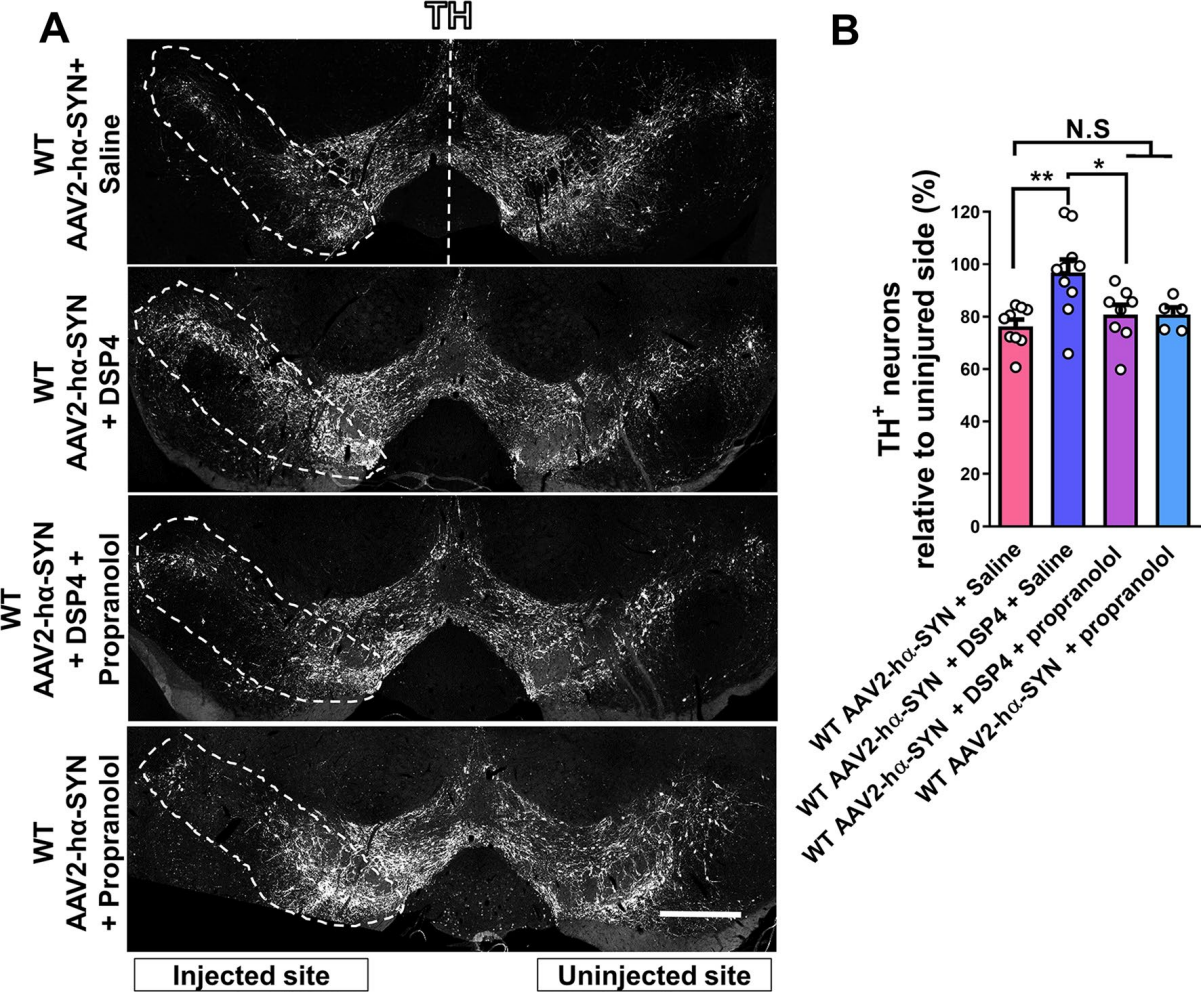
Blocking β-adrenergic receptors inhibits DSP-4-mediated dopaminergic neurons protection after hα-SYN overexpression. **A** Representative images of the SN showing dopaminergic neuron degeneration in the injected SN (TH, white) and **B** quantification of TH+ dopaminergic neurons in coronal sections of the SN 4 weeks after rAAV2–hα-SYN injection of WT mice in the different experimental conditions. Data is shown as mean values ± SEM, each dot represents one mouse, NS: not significant, *p < 0.05; **p < 0.01. One-way ANOVA followed by Tukey post hoc test. Scale bar = **A** 500 µm

DSP-4 is proposed to be a potent neurotoxin leading to depletion of brain NE and enhancing dopaminergic neuron degeneration in neurotoxin-based mouse models of PD [4–6]. However, this does not seem to apply in the hα-SYN virus-based mouse model of PD (Fig. 2). It has been reported that DSP-4 treatment leads to depletion of overall NE tissue levels in brain homogenates, which include both intracellular and extracellular levels of NE. However, studies using in vivo microdialysis to measure only extracellular NE levels after DSP-4 treatment have shown that NE extracellular levels in the brain are actually increased after DSP-4 treatment [27–29]. This suggests that DSP-4 may enhance extracellular NE levels and subsequent NE signaling, by blocking NET uptake of NE despite an overall reduction in total NE tissue content, which includes both intracellular and extracellular levels of NE. Notably, an increase in NE signaling in the brain has been reported to have neuroprotective effects in various CNS pathologies, including PD [12, 13, 16–18]. In particular, β2-AR signaling has been associated with a reduction in dopaminergic neuron degeneration in the MPTP and lipopolysaccharide (LPS) mouse model of PD [16, 18]. In addition, promoting β-1 signaling is shown to be neuroprotective in mouse model of Alzheimer’s diseases (AD) and Down's syndrome [20, 30, 31]. To test whether either of these two β-ARs could be involved in DSP-4-mediated protection of dopaminergic neurons in the SNpc, we used propranolol, a non-selective β-AR inhibitor, this antagonist blocks both β-1 and β-2 ARs [32]. Propranolol was given daily (10 mg/kg; IP) after rAAV2–hα-SYN injection in the SN of WT mice pretreated with DPS-4 or saline. We found that daily treatment of propranolol in DSP-4-treated WT mice inhibited dopaminergic neuron protection compared to only DSP-4-treated WT mice 4 weeks after hα-SYN overexpression (Fig. 3A, B). DSP-4 treatment alone showed significant protection of dopaminergic neurons compared to saline-treated WT mice after hα-SYN overexpression. There was no significant difference in dopaminergic neuron degeneration among saline-treated, propranolol + DSP-4-treated and only propranolol-treated WT mice 4 weeks after rAAV2–hα-SYN injection (Fig. 3A, B). The presence of hα-SYN in dopaminergic neurons was confirmed in all experimental conditions using an antibody specific for hα-SYN (Additional file 1: Fig. S3). Overall, these data suggest that DSP-4-mediated protection of dopaminergic neurons in the SN involves signaling through the β-ARs in the context of hα-SYN-mediated neurotoxicity.

### β-2 but not a β-1 AR agonist protects dopaminergic neurons in the SN from hα-SYN-mediated neurotoxicity

**Fig. 4:**
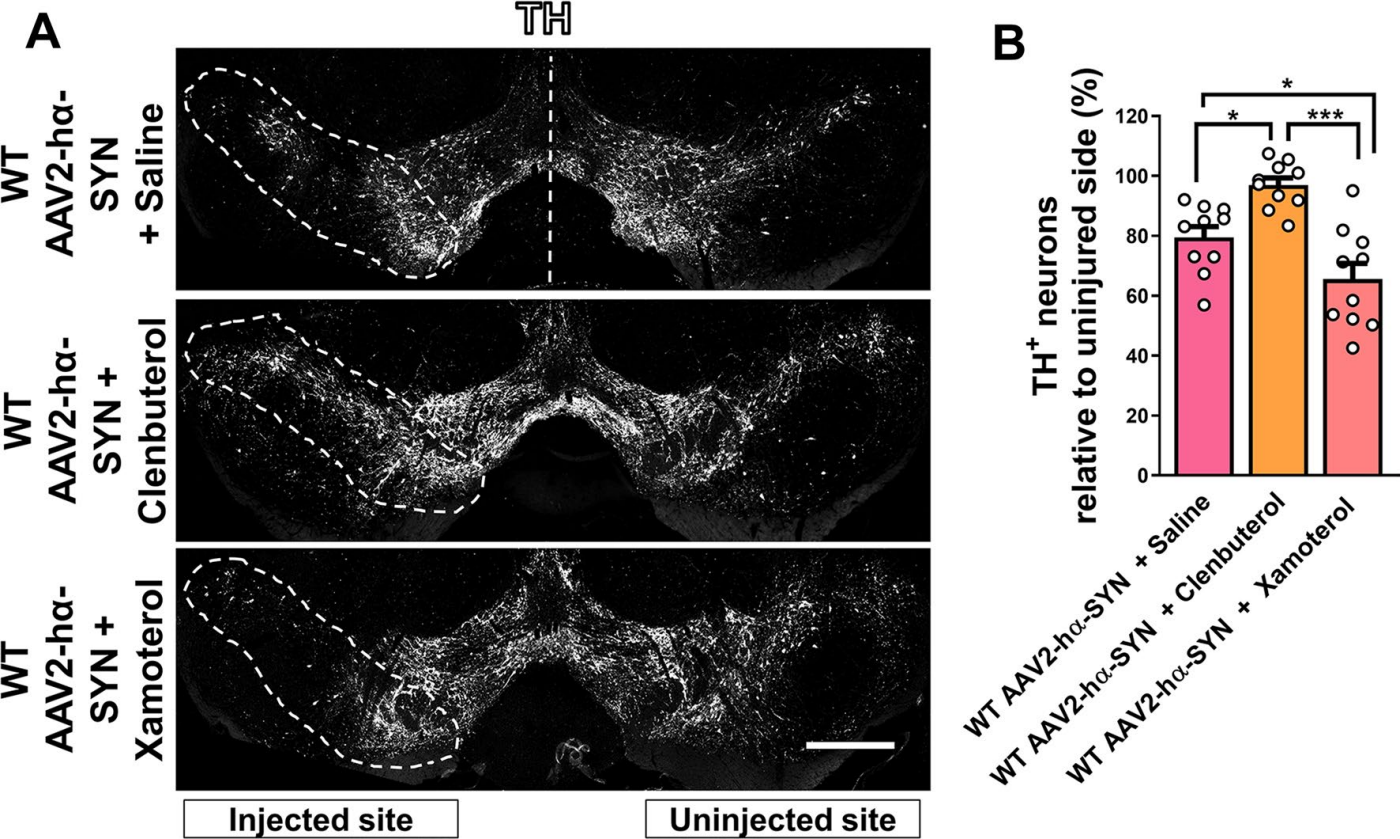
β-2 but not a β-1 AR agonist protects dopaminergic neurons in the SN from hα-SYN-mediated neurotoxicity. **A** Representative images of the SN showing dopaminergic neurons degeneration in the injected SN (TH, white) and **B** quantification of TH+ dopaminergic neurons in coronal sections of the SN 4 weeks after rAAV2–hα-SYN injection in WT mice treated daily with saline, Xamoterol or Clenbuterol. Data are shown as mean values ± SEM, each dot represents one mouse, NS: not significant, *p < 0.05; ***p < 0.001. One-way ANOVA followed by Tukey post hoc test. Scale bar = 500 µm

Based on the observation that DSP-4 protects dopaminergic neuron degeneration presumably by increasing β-ARs signaling (Fig. 3). We wanted to explore whether β-1 or β-2 AR signaling was driving dopaminergic neuron protection in the context of hα-SYN-mediated neurotoxicity. To test this, we used Xamoterol and Clenbuterol, β-1 and β-2 AR agonists, respectively [33, 34]. Daily treatment of Clenbuterol (10 mg/kg; IP) showed significant protection of dopaminergic neurons compared to saline and Xamoterol-treated WT mice 4 weeks after overexpression of hα-SYN (Fig. 4A, B). Interestingly, daily treatment of Xamoterol (3 mg/kg; IP) showed a significant increase of dopaminergic neuron degeneration compared to saline-treated WT mice 4 weeks after rAAV2–hα-SYN injection, Fig. 4A, B). The presence of hα-SYN in dopaminergic neurons was confirmed in all experimental conditions using an antibody specific for hα-SYN (Additional file 1: Fig. S4). Overall, these data suggest that signaling by β-1 and β-2 AR may have opposing effects in the context of hα-SYN-mediated neurotoxicity, since promoting β-2 AR signaling is neuroprotective, whereas β-1 AR signaling is neurodegenerative.

### Opposite effects of β-1 and β-2 AR signaling in microglia activation after hα-SYN overexpression in the SN

**Fig. 5:**
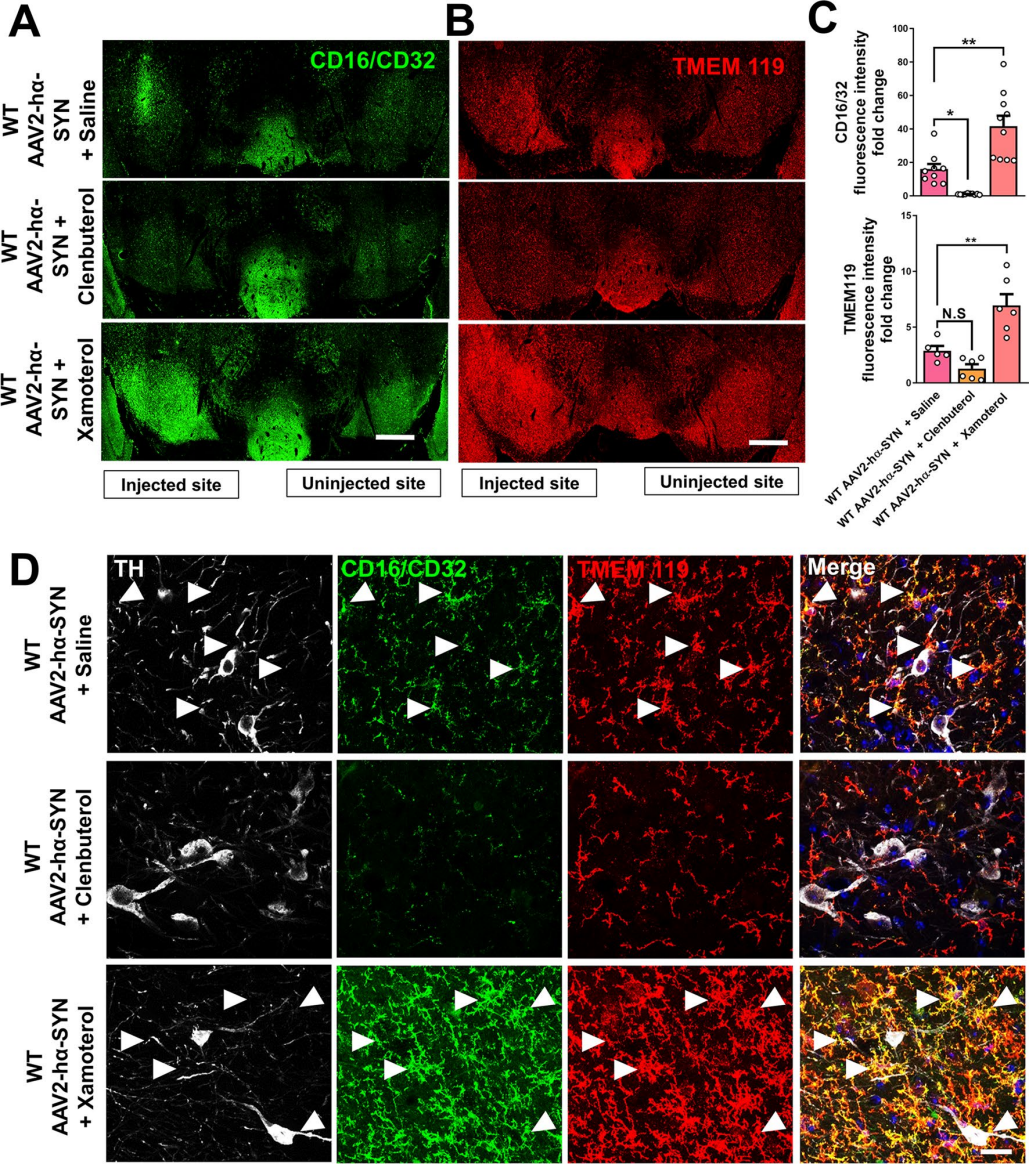
Opposing effects of β-1 and β-2 AR signaling in microglia activation after hα-SYN overexpression. Coronal sections of the SN showing **A** CD16/32 and **B** TMEM119 staining and **C** their respective quantifications indicating fluorescence intensity fold change relative to saline-treated WT mice 4 weeks after rAAV2–hα-SYN injection in clenbuterol and xamoterol treated WT mice. **D** Confocal images of the SN showing TMEM119 (red), TH (white) and CD16/32 (green) staining 4 weeks after rAAV2–hα-SYN injection in WT mice treated with saline, clenbuterol or xamoterol daily. Arrows indicate TMEM119 and CD16/32 colocalization. Confocal images are shown as maximum intensity projections. Data are shown as mean values ± SEM, each dot represents one mouse, NS: not significant, *p < 0.05; **p < 0.01. One-way ANOVA followed by Tukey post hoc test. Scale bar = **A**, **B** 500 µm, **D** 20 µm

In PD, β-2 AR signaling is associated with a reduction in microglia activation in vivo and in vitro [18, 35, 36]. In human and animal models of PD, microglia activation is suggested to contribute to the degeneration of dopaminergic neurons in the SNpc [37]. To explore whether Clenbuterol-mediated protection of dopaminergic neurons after hα-SYN overexpression was associated with a reduction in microglia activation in the SN, we stained coronal brain sections of the SN with CD16/32, TMEM-119 and TH 4 weeks after rAAV2–hα-SYN injection. CD16/32 was used as a proinflammatory marker of microglia [38, 39] and TMEM-119 was used as a marker of microglia [40]. We observed that Clenbuterol treatment significantly reduced CD16/32 immunoreactivity in the SN compared to saline-treated WT mice, whereas Xamoterol treatment significantly increased both, CD16/32 and TMEM-119 immunoreactivity compared to saline-treated WT mice 4 weeks after rAAV2–hα-SYN injection (Fig. 5A–C). Clenbuterol treatment shows a non-significant trend towards the reduction of TMEM-119 immunoreactivity compared to saline-treated wild-type mice (Fig. 5A–C). Confocal images showed that pro-inflammatory microglia (CD16/32+ and TMEM119+ cells) were in close contact with dopaminergic neuron cell bodies and axons in saline-treated and Xamoterol-treated WT mice but not in Clenbuterol-treated WT mice 4 weeks after rAAV2–hα-SYN injection (Fig. 5D). These data suggest that promoting β-2 AR signaling reduces proinflammatory microglia, whereas promoting β-1 AR signaling enhances microglia proinflammatory response in the context of hα-SYN-mediated damage.

### β-2 AR agonist reduce T-cell infiltration and rescues behavioral deficits after hα-SYN overexpression in the SN

**Fig. 6:**
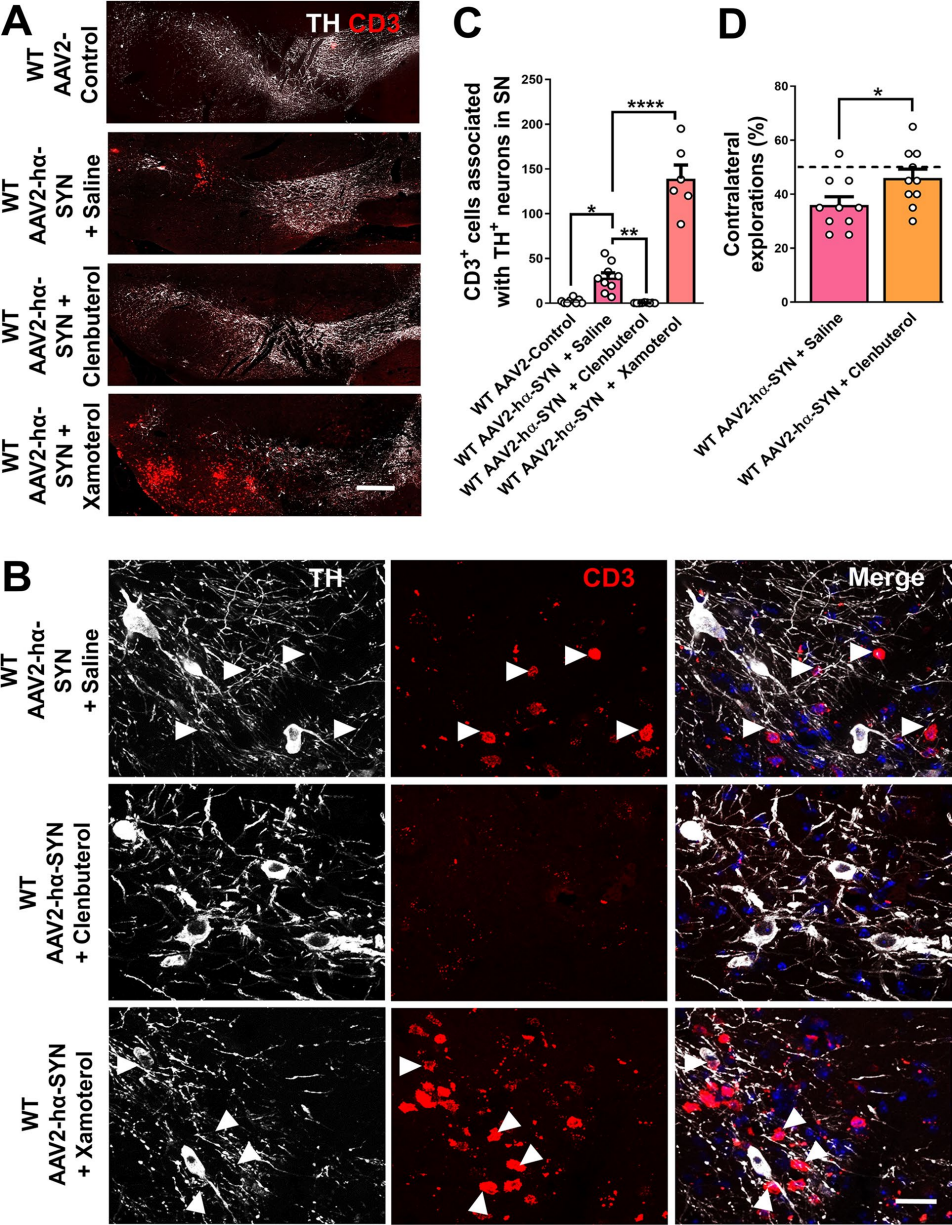
Clenbuterol reduces T-cell infiltration and rescues behavioral deficits after hα-SYN overexpression in the SN. **A** Representative images of the SN showing CD3 (red) and TH (white) staining and **B** confocal close up showing CD3+ cells in contact with TH cell bodies and axons (arrows). **C** Quantification of CD3+ T cells associated with the SN 4 weeks after rAAV2-control or rAAV2–hα-SYN injection in WT mice treated with saline, Clenbuterol or Xamoterol daily. **D** Quantification of sensorimotor bias in a corridor task 4 weeks after rAAV2–hα-SYN injection in WT treated with saline or clenbuterol daily. Black horizontal dash line represents the expected % of contralateral explorations in an uninjured WT mouse. Confocal images are shown as maximum intensity projections. Data are shown as mean values ± SEM, each dot represents one mouse, N.S: not significant, *p < 0.05; **p < 0.01; ****p < 0.0001. One-way ANOVA followed by Tukey post hoc test; two-tailed *t* test. Scale bar = **A**, **B** 500 µm, **D** 20 µm

In human patients and mouse models of PD, T-cell infiltration has been associated with dopaminergic neuron degeneration and PD pathology [41]. In the hα-SYN-based model of PD, T-cell infiltration is proposed to be a necessary step to induce dopaminergic neuron degeneration in the SNpc [42, 43]. To test if β-1 or β-2 AR signaling using xamoterol and clenbuterol, respectively, affected T-cell infiltration in the SN 4 weeks after rAAV2–hα-SYN injection, we stained coronal sections of the SN with TH and the T-cell marker, CD3. The injection of rAAV2-control into the SN did not result in T-cell infiltration in the SN, suggesting that the mechanical injury caused by the injection alone is not sufficient to allow T-cell extravasation in this PD mouse model (Fig. 6A, B). In addition, we found that clenbuterol treatment significantly reduced the infiltration of T-cell associated with dopaminergic neurons cell bodies and axons in the SN compared to saline-treated WT mice 4 weeks after rAAV2–hα-SYN injection (Fig. 6A–C). In contrast, Xamoterol treatment significantly increased T-cell infiltration compared to saline-treated WT mice 4 weeks after rAAV2–hα-SYN injection (Fig. 6A–C). Infiltration of T-cells into the brain may suggest damage of the BBB in the hα-SYN mouse model of PD; based on this, we explored the extent of BBB damage after hα-SYN overexpression in WT mice treated with Xamoterol and Clenbuterol. We found that Xamoterol significantly increases BBB damage compared with saline and Clenbuterol-treated WT mice 4 weeks after rAAV2–hα-SYN injection (Additional file 1: Fig. S5). BBB opening was measured by extravasation of mouse IgG into the brain parenchyma. To assess whether the neuroprotection associated with Clenbuterol treatment rescues behavioral deficits after rAAV2–hα-SYN injection, a lateralized sensory-motor integration test was used. This behavioral test measures the lateralized bias of exploring sugar pellets in a corridor task and it has been shown to be sensitive in detecting partial unilateral dopaminergic neuron damage [22, 44]. Clenbuterol treatment prevented the behavioral deficit in WT mice 4 weeks after overexpression of hα-SYN compared to saline-treated WT mice (Fig. 6D). Overall, our data showed that stimulation of the β-2 AR using clenbuterol reduced neuroinflammation and protected dopaminergic neuron degeneration, whereas stimulation of the β-1 AR using xamoterol promoted neuroinflammation and dopaminergic neuron degeneration in the context of hα-SYN-mediated neurotoxicity. This suggests that a selective β-2 AR agonist but not a β-1 AR agonist may be preferred as a possible treatment in PD.

## Discussion

NE regulates different physiological and pathological functions in the CNS, the NE system is affected in PD patients, and β2-AR signaling has been associated with a reduction in PD risk, whereas inhibition of β-AR signaling has been associated with an increase in PD risk. However, the role of NE signaling on dopaminergic neuron survival in the SNpc has not been yet elucidated. In this paper, we used a 6OHDA-based and hα-SYN-based model of PD to explore the effects of DSP-4 in dopaminergic neuron survival with the goal to better understand the role of NE signaling in PD pathology and to identify novel therapeutic approaches involving the NE system in PD. We found that DSP-4 pretreatment has different effects on dopaminergic neuron survival depending on the mouse model of PD used. In the 6OHDA PD model, DSP-4 increased dopaminergic neuron degeneration whereas, in the hα-SYN model of PD, DSP-4 treatment protected dopaminergic neurons. In both cases, DSP-4 interaction with NET was necessary to elicit their respective responses. Blocking the β-ARs using propranolol, inhibited DSP-4-mediated protection of dopaminergic neurons in the context of hα-SYN-mediated neurotoxicity. Finally, we identified that β2-AR signaling is a likely therapeutic target in PD that reduced microglial activation, T-cell infiltration, and dopaminergic neuron degeneration. In contrast, β1-AR signaling might be deleterious since xamoterol treatment increased neuroinflammation and dopaminergic neuron degeneration in the context of hα-SYN-mediated neurotoxicity.

We found that DSP-4 pretreatment protected dopaminergic neurons in the SN after hα-SYN overexpression. Our data conflicts with previous published studies in different PD mouse models PD [4–6, 45] and in other neurodegenerative diseases [46, 47]. These studies showed that DSP-4 pretreatment generally is associated with an increase in inflammation and neuronal degeneration. The DSP-4 dose used in this study is considered standard to elicit a robust depletion of NE tissue levels in the brain (single dose of 50 mg/kg; I.P) [19]. DSP-4 treatment is known to deplete NE tissue levels in brain homogenates, which measures both intracellular and extracellular levels of NE. However, it is not fully understood how DSP-4 specifically affects extracellular NE levels in the brain. While it is assumed that extracellular NE levels decrease due to the overall depletion of NE tissue levels observed in brain homogenates, some studies have challenged this assumption by showing that DSP-4 treatment can actually increase extracellular NE levels in the brain, as measured by in vivo microdialysis [19, 27–29]. This could explain our results showing DSP-4-mediated protection in the context of hα-SYN mediated neurotoxicity. Since DSP-4 treatment could increase extracellular NE levels and subsequent signaling in the brain which has been associated with a reduction in neuroinflammation and neuronal degeneration in vitro and in vivo [48, 49]. This hypothesis is supported by the observation that blocking the β-ARs using propranolol, reverses DSP-4 protection of dopaminergic neurons after hα-SYN overexpression. However, further investigation is needed regarding the effects of DSP-4 treatment on extracellular NE levels in the brain and its role in modulating neuroinflammation and neuronal survival in different neurodegenerative diseases. Although, we found that DSP-4 protects from hα-SYN-mediated neurotoxicity, a recent study using a hα-SYN transgenic mouse model of PD shows that DSP-4 treatment failed to protect or enhance dopaminergic neuron degeneration in the SN compared to WT mice [45]. In this study, 13 months after DSP-4 treatment led to similar levels of dopaminergic neuron degeneration in the SN of both WT and hα-SYN transgenic mice [45]. This suggests that long term exposure to DSP-4 can lead to dopaminergic neuron degeneration in the SN in WT mice. The exact mechanism(s) of how DSP-4 may be leading to dopaminergic neuron degeneration in the SN is unknown.

A caveat with the hypothesis that increased extracellular NE might be driving DSP-4-mediated neuroprotection is that this fails to explain why DSP-4 pretreatment is not neuroprotective in other mouse models of PD, like the 6OHDA and LPS mouse model of PD [4–6]. A possibility that needs further investigation is that in these models of PD the expression of different ARs could be upregulated or downregulated favoring different NE signaling pathways that could exacerbate neurodegeneration after DSP-4 treatment. For instance, intraventricular injection of 6OHDA is reported to increase the density of β1-AR and to a lesser extent β2-AR in different brain regions, like the cortex and amygdala [50, 51]. Additionally, bilateral injection of 6OHDA in the SN induced the expression of β1-AR in the gastrointestinal system which is associated with gastrointestinal dysmotility a non-motor symptom in PD [52]. Considering that β1-AR signaling increased neuroinflammation and dopaminergic neuron degeneration in the hα-SYN mouse model of PD (Figs. 4, 5, 6), it is possible that 6OHDA injection in the striatum could change β-AR expression patterns in the brain favoring β1-AR signaling leading to dopaminergic neuron degeneration after DSP-4 pretreatment. Although β1-AR could be a candidate for this, other AR may also play a role, including α1, α2 or β3-AR. β1-AR signaling is also associated with neuroprotection in other neurological disorders [20, 30, 31]. However, it is unknown the mechanisms by which β1-AR signaling mediates neuroprotection or neurodegeneration in the brain and more studies are needed to assess the effect of β1-AR signaling in neuronal survival in other PD models.

To our knowledge, few studies have shown that β2-AR signaling is neuroprotective in neurotoxin mouse models of PD [16, 18, 35]. We found that in hα-SYN-mediated damage, stimulation of β2-AR by clenbuterol reduced neuroinflammation and neurodegeneration. The mechanism(s) driving dopaminergic neuron protection after increased β2-AR signaling and the cells involved are still unknown. In the neurotoxin-based model of PD, one group suggested that β2-AR signaling regulates mouse α-SYN expression leading to neuroprotection [16], while a different group suggest that β2-AR signaling directly reduced microglial activation [18, 35]. In our hα-SYN mouse model of PD, expression of hα-SYN is driven by the chicken beta actin (CBA) promoter and hα-SYN protein was present in dopaminergic neurons after clenbuterol treatment discarding the possibility that β2-AR signaling is affecting the expression of hα-SYN directly in our mouse model of PD. On the other hand, RNAseq data after overexpression of hα-SYN showed the upregulation of different genes associated with innate and adaptive immune response [43], suggesting that neuroinflammation may play an important role in dopaminergic neuron degeneration in the context of hα-SYN neurotoxicity. In addition, microglia and T-cell-mediated cell death is proposed to be play a significant role in dopaminergic neuron degeneration in hα-SYN mouse model of PD [42, 43]. We found that β2-AR signaling reduces pro-inflammatory microglia and T-cell infiltration in the SN. This suggests that enhancing β2-AR signaling downregulates the neuroinflammatory response after overexpression of hα-SYN which is consistent with previous reports showing β2-AR signaling reducing microglia activation in a neurotoxin-based model of PD [18, 35]. It is unknown which cell types the β2-AR is acting on leading to dopaminergic neuronal protection after rAAV2–hα-SYN injection. Neurons and different immune cells express β2-AR in the CNS. We found that β2-AR is expressed on TH+ neurons in vivo and others have reported expression of this receptor in microglia and T-cells. In vitro studies have shown that β2-AR signaling in all these cell types has been associated with reduction of proinflammatory markers and reduced neurotoxicity [35, 53]. However, the possible role of β2-AR signaling in these different cell types in mouse models of PD has not yet been explored.

To study the effects of DSP-4 and AR agonists in PD pathology, we used only male mice, this should be considered in interpreting our data, since sexual dimorphism has been identified in rats and mice regarding NE signaling in the brain [54, 55]. In addition, we only use a pharmacological approach in dissecting the possible mechanism involved in DSP-4-mediated protection of dopaminergic neurons in the context of hα-SYN damage and off-target effects of propranolol, clenbuterol and xamoterol could be confounding variables skewing our data interpretation. Future experiments should focus on confirming the role of β1-AR and β2-AR signaling in PD pathology with different complementary strategies. For instance, the use of chronic genetic models of PD-like hα-SYN transgenic mouse models will help assess the effects of chronic treatments with clenbuterol or xamoterol in AD pathology. This may give insights into the long-term effects of these drugs in the context of hα-SYN-mediated damage. In addition, mechanistic experiments using β1, β2 and β1/β2 double knock out mice in hα-SYN models of PD may be necessary to confirm the effect of clenbuterol and xamoterol in PD pathology since off-target drug effects are a possibility in our study.

## Conclusions

Using a hα-SYN mouse model of PD, we showed that DSP-4 pretreatment protects dopaminergic neuron degeneration, likely due to an increase in extracellular NE and signaling in the brain, since using propranolol a β-AR blocker prevented DSP-4-mediated neuroprotection. In addition, clenbuterol and not xamoterol treatment reduced neuroinflammation and dopaminergic neuron degeneration in the context of hα-SYN-mediated neurotoxicity. This suggests that specific β2-AR agonists like clenbuterol may be beneficial therapeutics in PD, whereas our data with the β1-AR agonist xamoterol indicates that β1-AR activation is associated with increased dopaminergic neuron degeneration in the SN, suggesting that the use of β1-AR agonists in PD patients may not be warranted.

## Supplementary Information

**Additional file 1.** Additional figures.

## Data Availability

All unique reagents generated in this study are available from the lead contact upon request and any additional information required to reanalyze the data reported in this paper is available from the lead contact upon reasonable request.

## Abbreviations

LC: Locus coeruleus
NE: Norepinephrine
SN: Substantia nigra
SNpc: Substantia nigra *pars compacta*
PD: Parkinson’s disease
AR: Adrenergic receptors
α-SYN: α-Synuclein
NET: Norepinephrine transporter
CNS: Central nervous system
6OHDA: 6-Hydroxydopamine
DSP-4: *N*-(2-Chloroethyl)-*N*-ethyl-2-bromobenzylamine hydrochloride
HPLC-ED: High-performance liquid chromatography with electrochemical detection
AD: Alzheimer’s diseases
LPS: Lipopolysaccharide
TH: Tyrosine Hydroxylase
