# Supplementary material for: Opposing effects of β-2 and β-1 adrenergic receptor signaling on neuroinflammation and dopaminergic neuron survival in α-synuclein-mediated neurotoxicity
Source: J Neuroinflammation. 2023 Mar 2;20:56. doi: 10.1186/s12974-023-02748-3 (PMC9983231; doi:10.1186/s12974-023-02748-3)
Supplement: Supplementary file 1 — Additional file 1. Additional figures. [file 12974_2023_2748_MOESM1_ESM.pdf]

Additional file 1

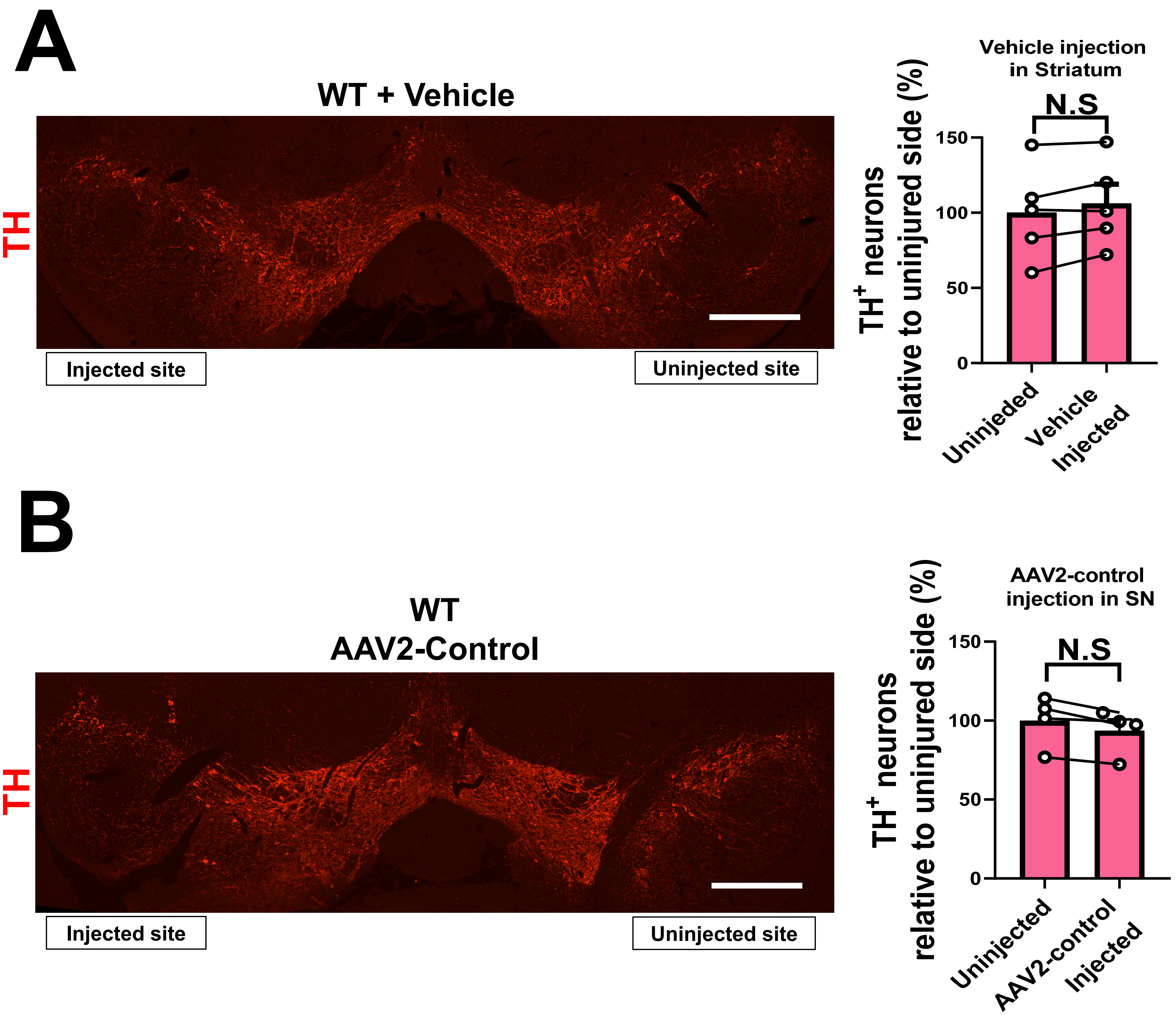

Figure S1. No effect in dopaminergic neuron degeneration after AAV2-Control injection in the SN or Vehicle into the striatum. Representative images of the SN showing TH staining in the SN confirming the presence of TH+ neurons in WT mice injected with vehicle into the Striatum (A) or AAV2-Control in the SN (B). (Right) Quantification of TH+ neurons in coronal sections of the SN 4 weeks after AAV2-Control in the SN or vehicle intro the Striatum in WT mice. Data is shown as mean values ± SEM, each dot represents one mouse, N.S=not significant. 2-tailed t-test. Scale bar= 500 µm.

Additional file 1

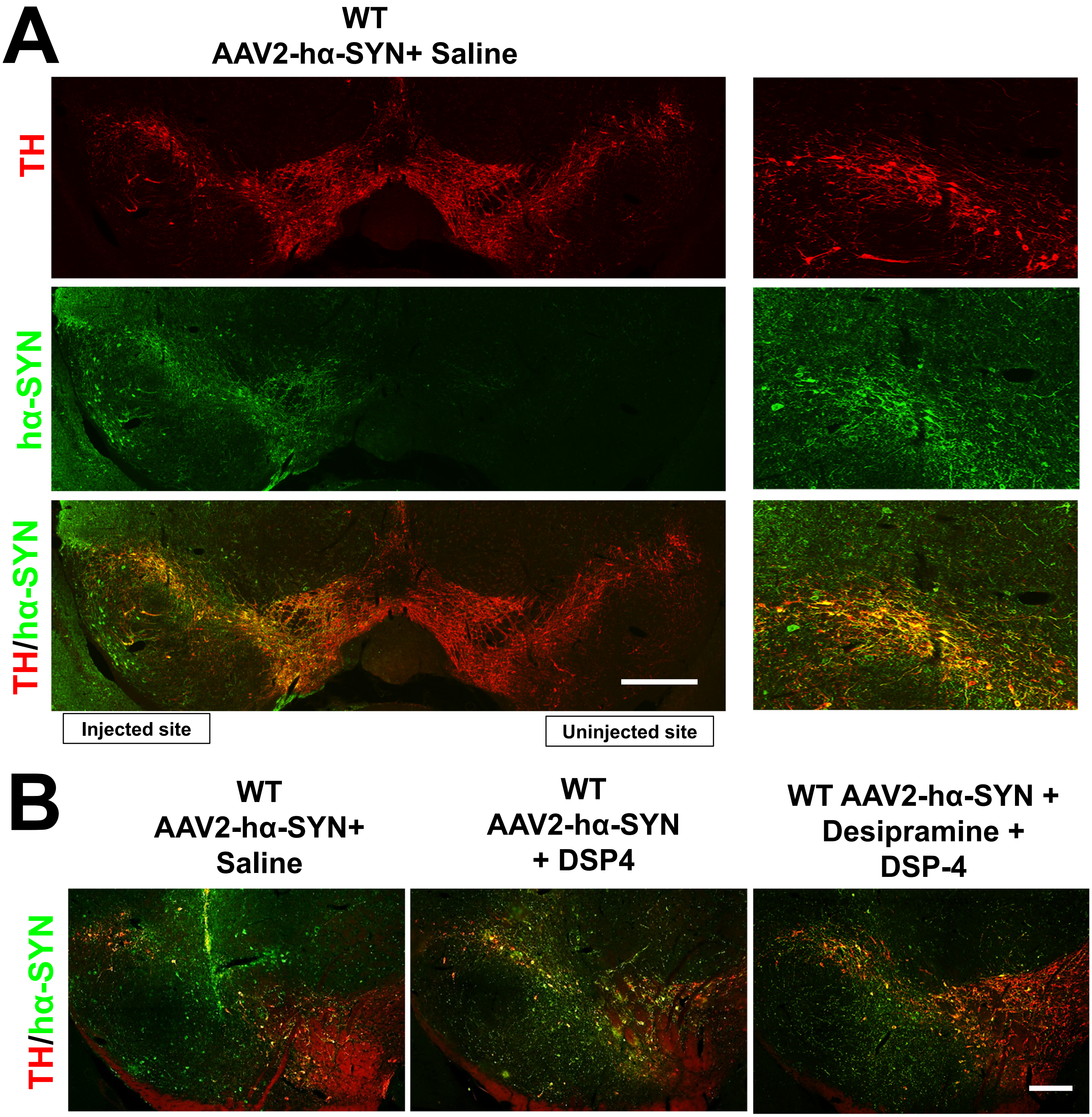

Figure S2. Confirmation of overexpression of hα-SYN in the SN after DSP-4 treatment. (A) Representative images of the SN showing TH (red) and hα-SYN (green) staining in the SN showing selectivity of hα-SYN antibody to the injected side of the SN; (Right) Close up of dopaminergic neurons. (B) Representative images of the SN showing TH (red) and hα-SYN (green) staining in the SN confirming hα-SYN expression in TH+ neurons in WT mice pretreated with Saline, DSP-4 or Desipramine + DSP-4.Yellow indicates colocalization of TH and hα-SYN in the SN. Scale bar= (A) 500 μm (B) 250 μm.

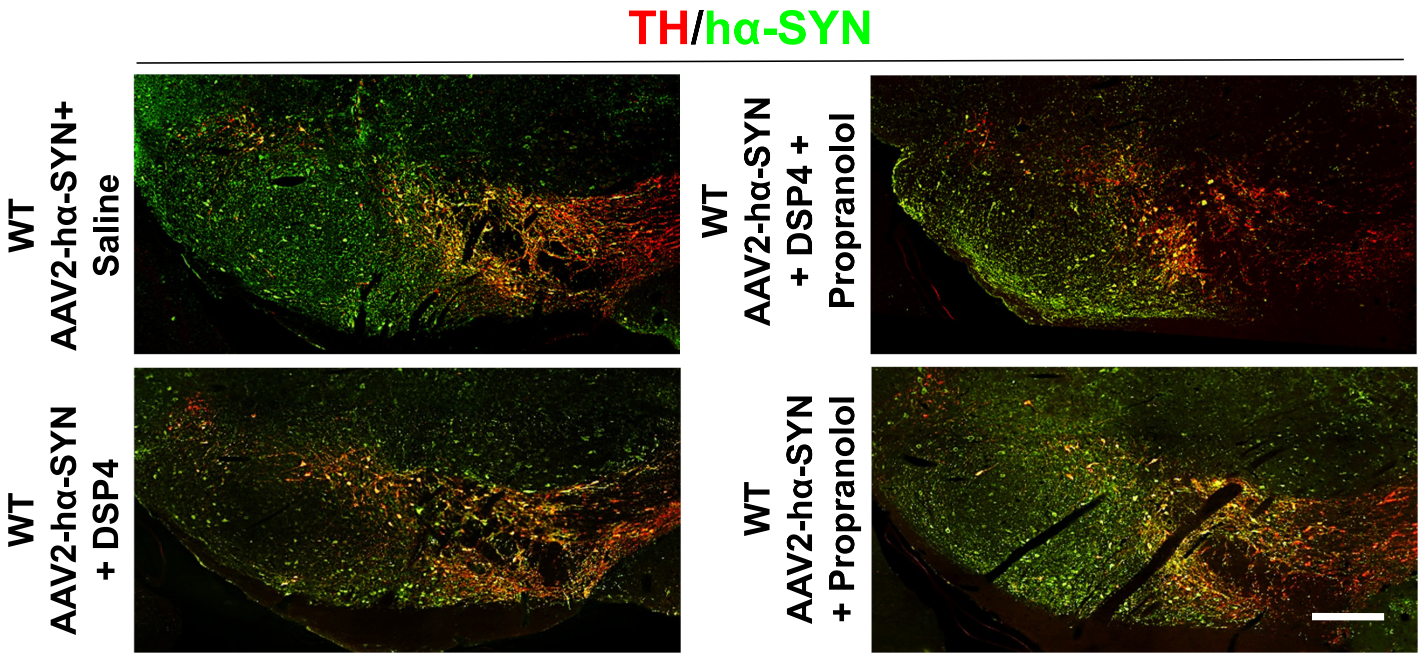

Figure S3. Confirmation of overexpression of hα-SYN in the SN after DSP-4 and propanolol treatment. Representative images of the SN showing TH (red) and hα-SYN (green) staining in the SN confirming hα-SYN expression in TH+ neurons in the different experimental conditions. Yellow indicates colocalization of TH and hα-SYN in the SN. Scale bar= 250 µm.

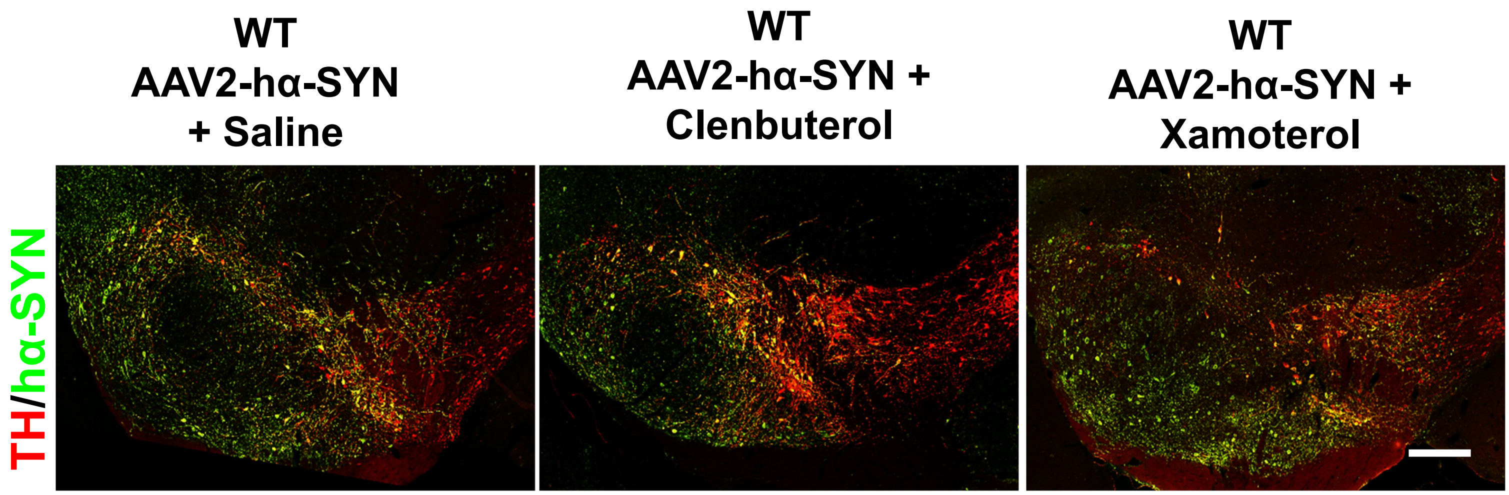

Figure S4. Confirmation of overexpression of hα-SYN in the SN after Clenbuterol and Xamoterol treatment. Representative images of the SN showing TH (red) and hα-SYN (green) staining in the SN confirming hα-SYN expression in TH+ neurons in WT mice treated daily with saline, Xamoterol or Clenbuterol. Yellow indicates colocalization of TH and hα-SYN in the SN. Scale bar= 250 μm.

.

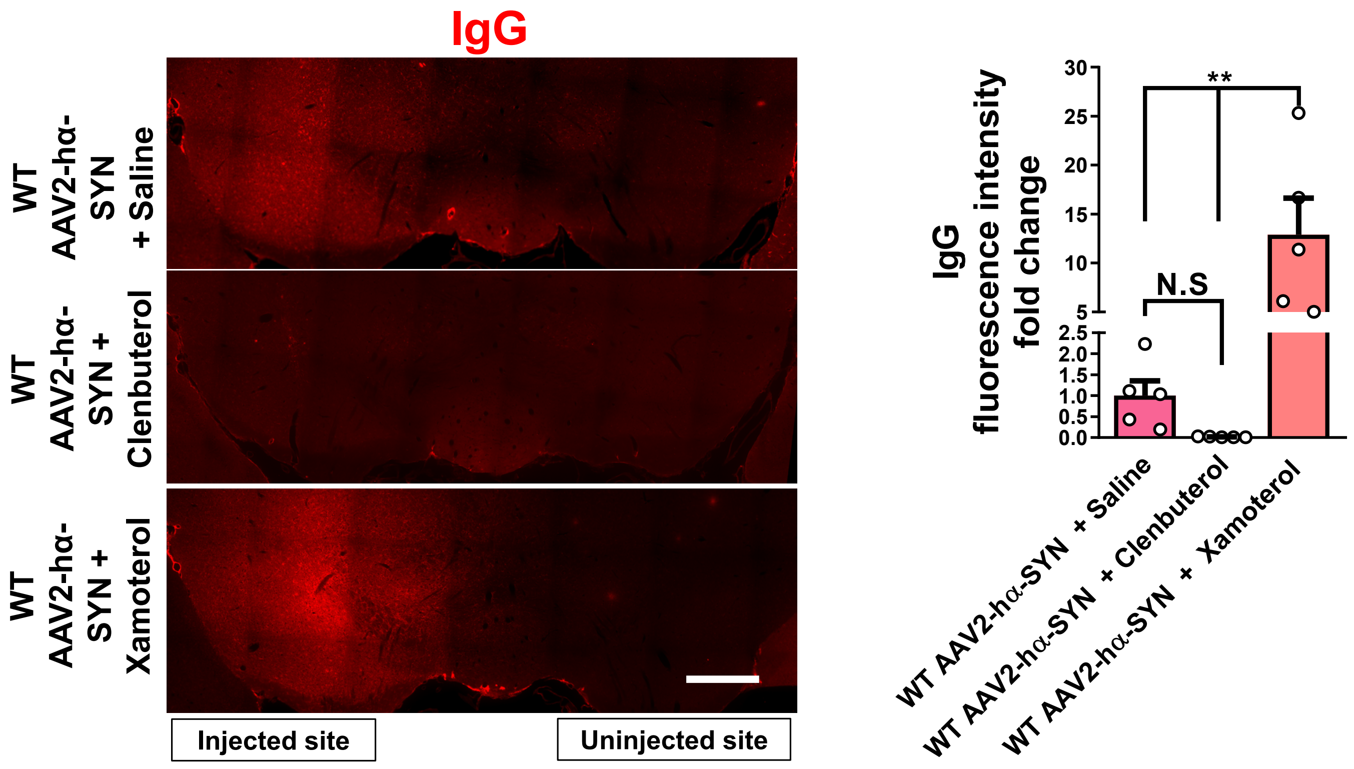

Figure S5. Clenbuterol reduces IgG extravasation compared to Xamoterol treatment after hα-SYN overexpression in the SN. Coronal sections of the SN showing IgG staining and quantification indicating fluorescence intensity fold change relative to saline-treated WT mice 4 weeks after rAAV2-hα-SYN injection in clenbuterol and xamoterol treated WT mice. Data is shown as mean values ± SEM, each dot represents one mouse, N.S=not significant, **$p<0.01$. 1-way ANOVA followed by Tukey post hoc test. Scale bar= 500 μm.
